# Diversification of Nitrogen Sources in Various Tundra Vegetation Types in the High Arctic

**DOI:** 10.1371/journal.pone.0136536

**Published:** 2015-09-16

**Authors:** Grzegorz Skrzypek, Bronisław Wojtuń, Dorota Richter, Dariusz Jakubas, Katarzyna Wojczulanis-Jakubas, Aleksandra Samecka-Cymerman

**Affiliations:** 1 West Australian Biogeochemistry Centre, School of Plant Biology, The University of Western Australia, Crawley, Western Australia, Australia; 2 Department of Ecology, Biogeochemistry and Environmental Protection, The University of Wrocław, Wrocław, Poland; 3 Department of Botany and Plant Ecology, The Wrocław University of Environmental and Life Sciences, Wrocław, Poland; 4 Department of Vertebrate Ecology and Zoology, The University of Gdańsk, Gdańsk, Poland; Institution and Department: Université de Sherbrooke, CANADA

## Abstract

Low nitrogen availability in the high Arctic represents a major constraint for plant growth, which limits the tundra capacity for carbon retention and determines tundra vegetation types. The limited terrestrial nitrogen (N) pool in the tundra is augmented significantly by nesting seabirds, such as the planktivorous Little Auk (*Alle alle*). Therefore, N delivered by these birds may significantly influence the N cycling in the tundra locally and the carbon budget more globally. Moreover, should these birds experience substantial negative environmental pressure associated with climate change, this will adversely influence the tundra N-budget. Hence, assessment of bird-originated N-input to the tundra is important for understanding biological cycles in polar regions. This study analyzed the stable nitrogen composition of the three main N-sources in the High Arctic and in numerous plants that access different N-pools in ten tundra vegetation types in an experimental catchment in Hornsund (Svalbard). The percentage of the total tundra N-pool provided by birds, ranged from 0–21% in Patterned-ground tundra to 100% in Ornithocoprophilous tundra. The total N-pool utilized by tundra plants in the studied catchment was built in 36% by birds, 38% by atmospheric deposition, and 26% by atmospheric N_2_-fixation. The stable nitrogen isotope mixing mass balance, in contrast to direct methods that measure actual deposition, indicates the ratio between the actual N-loads acquired by plants from different N-sources. Our results enhance our understanding of the importance of different N-sources in the Arctic tundra and the used methodological approach can be applied elsewhere.

## Introduction

Plant growth and tundra development in the High Arctic are limited by nitrogen (N) availability [1]. Consequently, in addition to the cold climate and short growing season, N deficiency is a major constraint for carbon (C) sequestration in soils in the High Arctic. Hence, the identification of the relative contributions of different N-sources to total available N is critical for understanding the tundra capacity for C retention. Three sources are the greatest contributors to the general N-pool available for plants in the Arctic: 1) atmospheric deposition (NO_x_, NH_x_); 2) primary N_2_-fixation from the atmosphere; and 3) bird feces deposition.

Atmospheric deposition is spatially distributed, more or less equally at the small catchment scale, and similar N-loads are delivered each year [2]. However, global anthropogenic N-input is progressively increasing and this form of N-deposition may vary seasonally depending on atmospheric conditions and distance to pollution emitters [2, 3]. In contrast to this type of deposition, atmospheric N_2_ fixation shows great spatial diversity that reflects vegetation mosaics and microtopography [4] and it directly depends on the presence of primary N-fixing organisms such as cyanobacteria (e.g., *Nostoc*, *Anabaena*, or *Calothrix*) [5, 6]. The N-input generated from N_2_ fixation does not change rapidly from year to year; however, it can be impacted by changes in the duration of snow cover persistence or the temperature of the growing season [4]. This source can be very significant and may constitute as much as 58–80% of the N-pool in Arctic ecosystems [4, 7, 8].

The third main N-source, bird feces, has an uneven distribution over time and space. Bird-delivered nutrients are deposited only during the short nesting period that occurs during the three summer months and coincides with the peak of the plant growing season [9]. The high loads of nutrients deposited in the areas adjacent to bird colonies are not fully used, as plant demands are frequently lower than the deposited supply. However, high N-loads allow growth of the much more N-demanding vascular plants. After the birds’ departure in autumn, the growing season terminates because of decreasing temperatures. The unused nutrients remain frozen until the following spring, when a significant portion of N is washed out by thawing snow. Nevertheless, the amount of nutrients supplied by bird feces in polar regions is much larger than from any other source [10].

The birds introduce these materials to the terrestrial ecosystem through their feces, carcasses, dead chicks, eggshells, food scraps, and feathers, considerably influencing the properties of the soil and vegetation [9, 11]. For all these reasons, large seabird colonies play a crucial role in the functioning of the ecosystem by initiating local growth of plants and increasing the concentrations of animals. The colonies increase the primary and secondary production, including the fueling of local production of phytoplankton in coastal waters neighboring their colonies [12]. They also increase the overall species diversity (summarized in [11]), by creating sites for foraging, hiding, and breeding by herbivores [12, 13]. The strength of this influence of the seabirds on the functioning of the local ecosystem can be seen with the example of the introduction of the Arctic Fox (*Vulpes lagopus*) to the Aleutian Islands, which induced substantial changes in tundra plant productivity and community structure. Fox predation on seabirds, considerably reduced the avian nutrient transport from the ocean to the land, thereby negatively affecting soil fertility and transforming more productive grasslands to less productive maritime tundra ecosystems [13].

One of the bird species that is likely a major contributor to the N-pool in the coastal Arctic tundra is the zooplanktivorous Little Auk (or Dovekie; *Alle alle*). With a population of >37 million pairs worldwide (reviewed in [14]), Little Auks form large colonies that fertilize tundra with great amounts of deposited feces [11]. This deposition represents a huge N-load (up to 14 g/m^2^/yr in Svalbard [15]) compared to atmospheric deposition (e.g., 0.07 g/m^2^/yr in Svalbard [2]) or atmospheric N_2_-fixation (e.g. general range for Arctic: 0.02–0.20 g/m^2^/yr [4]). However, the deposition is largely restricted to sites of bird colonies and to flight zones between the colonies and the coastline.

Although the Little Auk is currently the most abundant Arctic seabird, in the near future it may experience substantial environmental pressure due to climate-change related processes in marine and terrestrial ecosystems [11]. The major food component of the Little Auk diet in the region of Svalbard is the planktonic crustacean, *Calanus glacialis*, associated with cold Arctic waters. Therefore, the expected reduction in sea ice thickness and extent in the Arctic Ocean could have a negative influence on the foraging pattern of the Little Auk by restricting access to its preferred food type. This restriction can have a serious impact on the Little Auk’s energy budget and result in population reduction and a northward shift of the bird’s breeding range [11, 16]. Consequently, increases in environmental pressures on this species may change the tundra N-budget and C-sequestration as it is uncertain which bird species (and to what extent) may replace the Little Auk. Some recent studies suggest that Arctic ecosystems can be even more responsive to additions of N than to changes in temperature, light, or CO_2_ concentration [4]. Therefore, assessing the significance of bird-originated N-input for different types of tundra is of great importance [17].

Estimation of the contribution of N-loads from three major sources to the Arctic ecosystems continues to be a challenge, given the constraints associated with directly measured N-deposition and the very low N concentrations. Direct measurement of N-load deposition onto the ground surface provides information about the total pool available, but not about the amount that plants actually use compared to other N-sources. This basic approach does not usually reveal what portion of the N-loads delivered from different N-sources is bio-available and what is washed out by thawing snow and precipitation. For these reasons, indirect methods, such as analysis of stable nitrogen isotope composition (δ^15^N) of plants or snow green algae, are useful for evaluation of the actual N-sources contributing to plant growth.

The δ^15^N values of plants represent the δ^15^N values of the sources, but to some extent are modified due to physiological mechanisms within the plant [18, 19]. Consequently, the whole plant δ^15^N value for most plant species reflects the weighted mean of δ^15^N values of the contributing N-sources [20]. This is particularly relevant for N-limited Arctic systems, where nitrification is very restricted at <5°C [21]. Mineralization of organic matter usually leads to only small isotope fractionation of nitrogen (Δ^15^N<1‰) and the large isotope fractionations are actually caused by the nitrification of ammonium, not the initial conversion of organic N to ammonium [22]. Other processes, such as denitrification and ammonia volatilization, can cause significant isotope fractionation but require anoxic conditions. Therefore, the processes are largely restricted to bird colonies with very heavy loads of birds feces or deeper soil horizons not accessible to mosses [23, 24]. Even if fractionation occurs during changes in the N_org_-NH_4_-NO_3_ stable isotope composition of N-bearing compounds, it is likely to have limited influence on plant isotope composition outside birds colonies, where in very N-limited ecosystem most of nitrogen is quickly used or wash out [22]. Hence, the ratios between inputs from different N-sources to various types of N-limited tundra can be quantified if each of these N-sources has a distinct isotope signature.

The aim of this study was twofold: 1) to investigate the input from the three main N-sources (atmospheric deposition, primary N_2_-fixation from the atmosphere, and bird feces deposition) to plants in ten tundra vegetation types in an experimental catchment in Hornsund (Svalbard, High Arctic) using an indirect method based on the δ^15^N values of major N-sources; 2) to test the direct response of plant *δ* ^15^N to different bird N-loads.

## Materials and Methods

### Ethical statement

All birds were handled following the Governor of Svalbard research permit no. RIS-ID 4857/2011 (issued for DJ and KW-J), and none died or showed signs of suffering during the short feces samples collection. Plants were sampled following the Governor of Svalbard permit no. RIS-ID 3704/2011 (issued for BW). *Alle alle* birds are not endangered, however, they are protected by law of Svalbard. Similarly the collected plants are not endangered, however collected on the protect land; therefore, these research permits were obtained.

### Location of the study area and climate conditions

**Fig 1:**
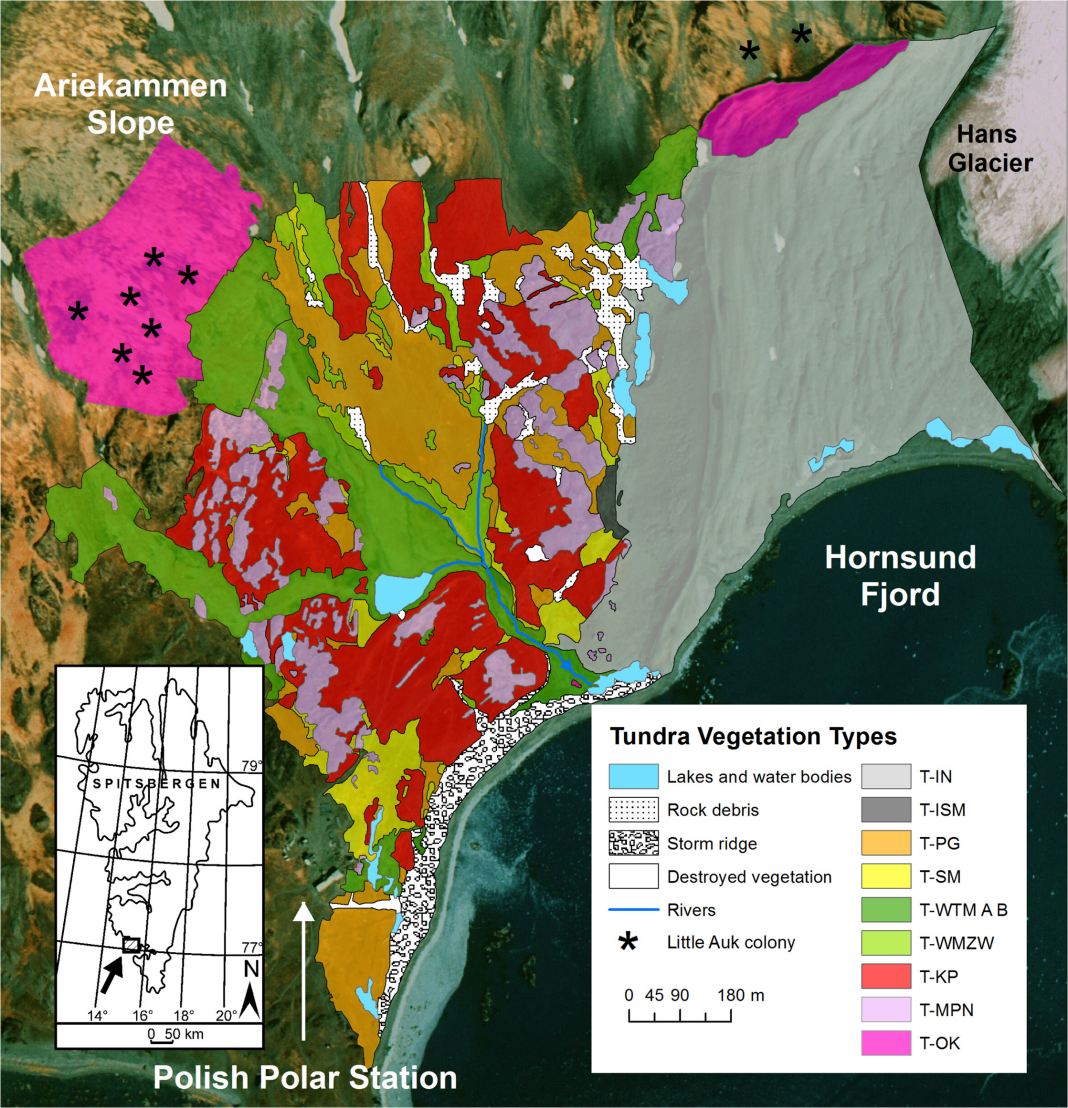
Distribution of different tundra vegetation types in the Fuglebekken catchment, Hornsund, Svalbard (77°00’N 15°30’E) based on orthoimage [25]. For tundra division details refer to Table 1.

The study was carried out in the Wedel Jarlsberg Land (77°00’N 15°30’E), within an unglaciated Fuglebekken catchment in the central part of the Fuglebergsletta abrasion-accumulation plain on the northern shore of Hornsund fjord in SW Spitsbergen, Svalbard (Fig 1, [25]). The climate on Spitsbergen is a polar and marine type. The annual mean air temperature in sampling year 2011 was –2.6°C, and was above the 30-year mean (-4.2°C for 1979–2010). The total annual precipitation in 2011 of 617 mm was significantly higher than the long-term annual mean (453 mm, 1979–2010). The period with air temperatures over 0°C is very short; therefore, the growing season is no longer than four months (June–September) (Figure A in S1 File). The thawing period usually begins in early June and ranges from 68 to 135 days, with an average of 92 days [26].

### Tundra vegetation types in the study area

The Fuglebekken catchment is characterized by a wide diversity of tundra types: ten major types of tundra were distinguished and mapped during an extensive botanical survey prior to sampling (Fig 1, Table 1 [27, 28]). The largest portion of the catchment (32.5%) is covered by Geophytic initial tundra (T-IN) developed on the lateral moraine adjacent to the Hansbreen glacier and characterized by sparse and floristically poor vegetation (Table 1).

**Table 1:** Tundra vegetation types in the study area, the Fuglebekken catchment (Hornsund, Svalbard), based on the field botanical survey. For location see Fig 1. Note that tundra T-WTM was subdivided into two categories depending on location (AB).

Symbol	Tundra type	Dominant species	Habitat and physiognomy	Presence of pimaryN_2_-fixers	% of total catchment area^	Bird-N assimilation ^^ [%]
T-IN	Geophytic initial	*Saxifraga oppositifolia* L., *Sanionia uncinata*, *Nostoc* spp.	Hyperskeletic Cryosols* on moraines. Sparse plant cover < 10%; low vegetation diversity.	Yes	32.5	12 (0–26)
T-ISM	Initial cyanobacteria-moss	*S*. *uncinata*, *Warnstorfia sarmentosa* (Wahlenb.) Hedenäs, *Nostoc* spp., *Tolypothrix tenuis*	Small, wet sandy and loamy marginal areas. Domination of cyanobacteria and mosses; 100% cover of the ground layer.	Yes (high?)	0.3	22 (0–46)
T-PG	Patterned-ground	*Nostoc* spp., *Dichothrix gypsophila*, *Anthelia juratzkana*, *S*. *uncinata*, *Bryum* sp.,	Turbic Cryosols* on flat surfaces or gentle slopes; wet sandy loam with rock fragments. Domination of cyanobacteria; 60–95% cover in the ground layer.	Yes (high?)	11.2	9 (0–21)
T-SM	Snowbed cyanobacteria-moss	*A*. *juratzkana*, *S*. *uncinata*, *Tolypothrix tenuis*, *Nostoc* spp., *Scytonema crustaceum*	Flat, wet places with gravel and sand; Haplic Cryosols*, from 70 to 100% cover of the ground; mostly small stands.	Yes (high?)	3.4	42 (19–62)
T-WTM A	Wet moss Just beneath *Alle alle* colony	*Starminergon stramineum* (Dicks. ex Brid.) Hedenäs, *S*. *uncinata*, *Aulacomnium palustre* (Hedw.) Schwägr., *Splachnum vasculosum* Hedw., *Tetraplodon mnioides*	Flat or gently sloping ground with permanent standing water; Hyperskeletic Cryosols*. Species-poor assemblage with mosses predominance in a form of dense, flat and extensive mats.	No	2.5	100 (100–100)
T-WTM B	Wet moss ca. 500 m from *Alle alle* colony	*Starminergon stramineum*, *S*. *uncinata*, *A*. *palustre*	Flat or gently sloping ground with permanent standing water; Hyperskeletic Cryosols*. Species-poor assemblage with mosses predominance in a form of dense, flat and extensive mats.	No	10.3	83 (67–100)
T-WMZW	Flow water wet moss	*W*. *sarmentosa*, *S*. *uncinata*, *Tolypothrix tenuis*	Wettest areas along melt-water runnels with flowing water. Masses or soft wet and spongy saturated small carpets.	Yes	2.9	28 (2–52)
T-KP	Lichen-prostrate shrub	*Cetrariella delisei*, *Ochrolechia frigida* (Sw.) Lynge, *Polytrichastrum alpinum*, *Salix polaris*	Dry locations on flat or gently slopes, well-drained ground with fine gravel and small pebbles; Haplic Cryosols*. Physiognomy is determined by lichens with frequent association of mosses and polar willow; cover large expenses on plains.	Yes (limited?)	17.0	27 (0–51)
T-MPN	Epilithic moss-lichen	*Racomitrium lanuginosum*, *Cetraria islandica* (L.) Ach., *Cladonia mitis* Sandst., *S*. *polaris*	Dry locations of acid rocks outcrops; Lithic Leptosols*. Predominance of *R*. *lanuginosum* which forms large mats; very dry and ombrophilous community.	Yes (limited?)	9.2	23 (0–48)
T-OK	Ornithocoprophilous	*Cerastium arcticum* Lange, *Poa alpine* L., *S*. *polaris*, *S*. *uncinata*, *Plagiomnium ellipticum* (Brid.) T.J.Kop., *Tetraplodon mnioides*	On slopes with a southern exposure, among rocky debris; under strong influence of the Little Auk (*Alle alle)* colony; Leptic Regosols (Ornithic)*. Vegetation forms two layers: compact (60–90%) vascular plants and cryptogams (20–70%); floristically the richest assemblage (*ca* 25 species).	No	10.7	100 (100–100)

* Soils and tundra classification after Szymański et al. [28].

^ The total catchment area is 1,385,939 m^2^ and tundra covers 1,282,195 m^2^ (excluding not vegetated areas, e.g. moraine and lakes).

^^ Bird-N [%] given as percentage of N assimilated from this source in relation to two other sources (N_2_-fixation and atmospheric deposition), in brackets are given possible ranges (minimum and maximum) depending on two other sources.

Initial cyanobacteria-moss tundra (T-ISM) is confined to wet depressions adjoining the lateral moraine. Patterned-ground tundra (T-PG) is related to areas with strong cryogenic processes and micro-relief forms. The central part of each relief, composed of fine and moist mineral material, is colonized by cyanobacteria and bryophyte crusts, and is surrounded by sorted circles of dry stones supporting mainly *Cetrariella delisei* (Bory ex Schaerer) Kärnefelt & Thell. Snow bed cyanobacteria-moss tundra (T-SM), with a predominance of cyanobacteria-*Anthelia juratzkana* (Limpr.) Trev. crusts and small mats of mosses, occurs in localities where snow collects in large amounts and thaws late. Permanently moist or wet habitats are vegetated by moss communities of Wet moss tundra (T-WTM) and Flow water wet moss tundra (T-WMZW). The T-WTM type is divided into two subgroups (A and B) depending on the distance from bird colonies. Dry and well-drained areas of the catchment are vegetated by Lichen-prostrate shrub tundra (T-KP) and Epilithic moss-lichen tundra (T-MPN). Sites with high accumulations of seabird feces are covered by Ornithocoprophilous tundra (T-OK) developed on steep slopes usually between 50 and 200 m a.s.l. The plants occurring at T-OK attain a much greater size and cover when compared to similar adjunct locations (Table 1). The nomenclature used for vascular plants follows Elven and Elvebakk [29], nomenclature for mosses follows Hill et al. [30], nomenclature for hepatics follows Schumacker and Váňa [31], and nomenclature for the lichens follows Wirth et al. [32].

### Sampling strategy

The sampling strategy was designed to obtain representative *δ* ^15^N data for 1) each of ten types of tundra, 2) three major N-sources, and 3) the altitudinal N deposition gradient with respect to the distance from the Little Auk colonies. More than 270 samples of plants, bird feces, soil, and water were collected during the 2011 growing/breeding season across the studied Fuglebekken catchment (for details, see S3 File and S1–S3 Tables) and analyzed for *δ* ^15^N.

Plant and soil samples from ten types of tundra were collected as three replicates from randomly selected locations representing the same type of tundra (3×10 = 30). Widespread species across different types of tundra included three mosses, *Sanionia uncinata* (Hedw.) Loeske (SAN), *Racomitrium lanuginosum* (Hedw.) Brid. (RAC) and *Polytrichastrum alpinum* (Hedw.) G.L.Sm. (POL), and dwarf willow, *Salix polaris* Wahlenb. (SLX). Samples of these four species, along with the plant species typical for each type of tundra, were collected, if available, from each sampling location (see Table 1). Two moss species (SAN and RAC) were particularly targeted because their nutrient acquisition relates to their pattern of water uptake [33]. These two species are ectohydric and take up N with water from precipitation [34]. In contrast, *Polytrichastrum alpinum* (*Polytrichospida*) is endohydric and transports water up from the underlying soil [35]. Direct N-uptake from soil was confirmed for moss species [36]; however, the lack of true roots in *Polytrichospida* limits its soil nutrient acquisition to a passive mode [37]. *Salix polaris* is a vascular plant with a well-developed root system capable of actively transporting N from deeper soil horizons that are not accessible to mosses. However, in Arctic conditions, the depth of root penetration rarely exceeds a few centimeters below ground level [38]. *S*. *polaris* also may be ectomycorrhizal, giving it access to an accessing additional N-pool [39]. Hence, the stable nitrogen isotope composition of *S*. *polaris* may not truthfully reflect the signatures of original N-sources contributing to the tundra, but may also represent recycled N from the soil.

**Fig 2:**
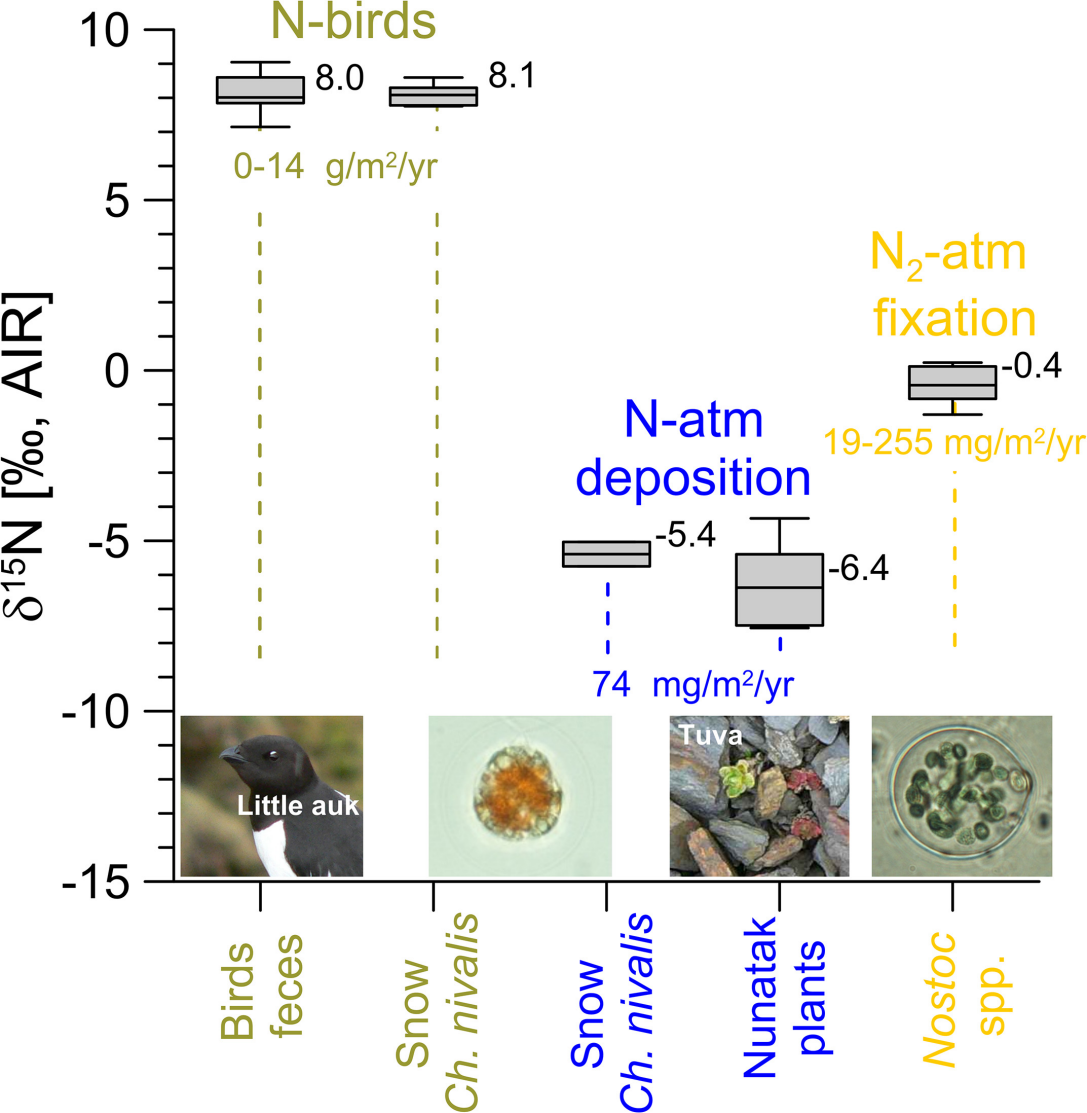
General range of the stable nitrogen isotope composition of the three major N-sources available for tundra plants: i) bird feces (N-birds), analyzed in fresh bird feces and the cryophilic alga *Chlamydomonas nivalis*; ii) N-atmospheric deposition, analyzed in *C*. *nivalis* at sites away from the bird colonies and mosses from nunatak Tuva; iii) N_2_ primary fixation, analyzed in the N-fixing cyanobacteria *Nostoc* sp. Bird N-loads were recalculated from Zwolicki et al. [15], atmospheric N-deposition were based on Kühnel et al. [2] and atmospheric N_2_-fixation as measured for Arctic by Chapin and Bledsoe [40]. Photos by G. Skrzypek and D. Richter.

Three major N-sources for tundra plants were sampled across the catchment and from surrounding locations (Fig 2). Fresh Little Auk feces were collected over a three-week period directly from adult birds while handling them for purposes of another project. Because the amount of feces from a single individual was small, samples from 1–6 birds were pooled together in sampling vials. In total, 17 samples were obtained from 72 different individuals. Because an uncertainty exists regarding the extent to which bird feces reflect the actual *δ* ^15^N of the NO_3_/NH_4_/N_org_ used by plants, indirect methods of determination of *δ* ^15^N in the bioavailable N-pool were used. For indirect methods, *Chlamydomonas nivalis* and mosses exposed primarily to one N-source only were used for determination of δ^15^N of this source. This approach results in determination of a weighted mean signature that reflects the possible ratio between different chemical forms of N in each source.

The cryophilic green alga, *C*. *nivalis*, was collected from the snow in three locations near the Little Auk colonies. *C*. *nivalis* does not have the ability to fix atmospheric nitrogen and therefore relies on N deposited within the snow. *C*. *nivalis* was also used for tracing atmospheric N-deposition, as direct stable nitrogen isotope analyses of N in rain and snow were not successful due to the extremely low concentrations. The δ^15^N of atmospheric deposition was obtained by collecting two samples of snow with *C*. *nivalis* from higher elevations above the bird colonies, where the expected input from the birds was likely to be marginal. *Polytrichastrum* and *Racomitrium* mosses growing on mineral soils were also collected from three locations on the Tuva nunatak, located ~5 km inland, where bird visits were very limited. Finally, δ^15^N associated with primary N_2_-fixation from atmosphere [40] was obtained by sampling the thallus and heterocysts of *Nostoc* (n = 12). In addition, 30 samples of water, including rain, snow, and subsurface water from piezometers located in wet types of tundra were collected across the study sites for *δ* ^15^N and *δ* ^18^O and for NH_4_^+^/NO_3_^–^ analyses. The *δ* ^18^O value was used to distinguish the original atmospheric from biologically reprocessed NO_3_.

**Fig 3:**
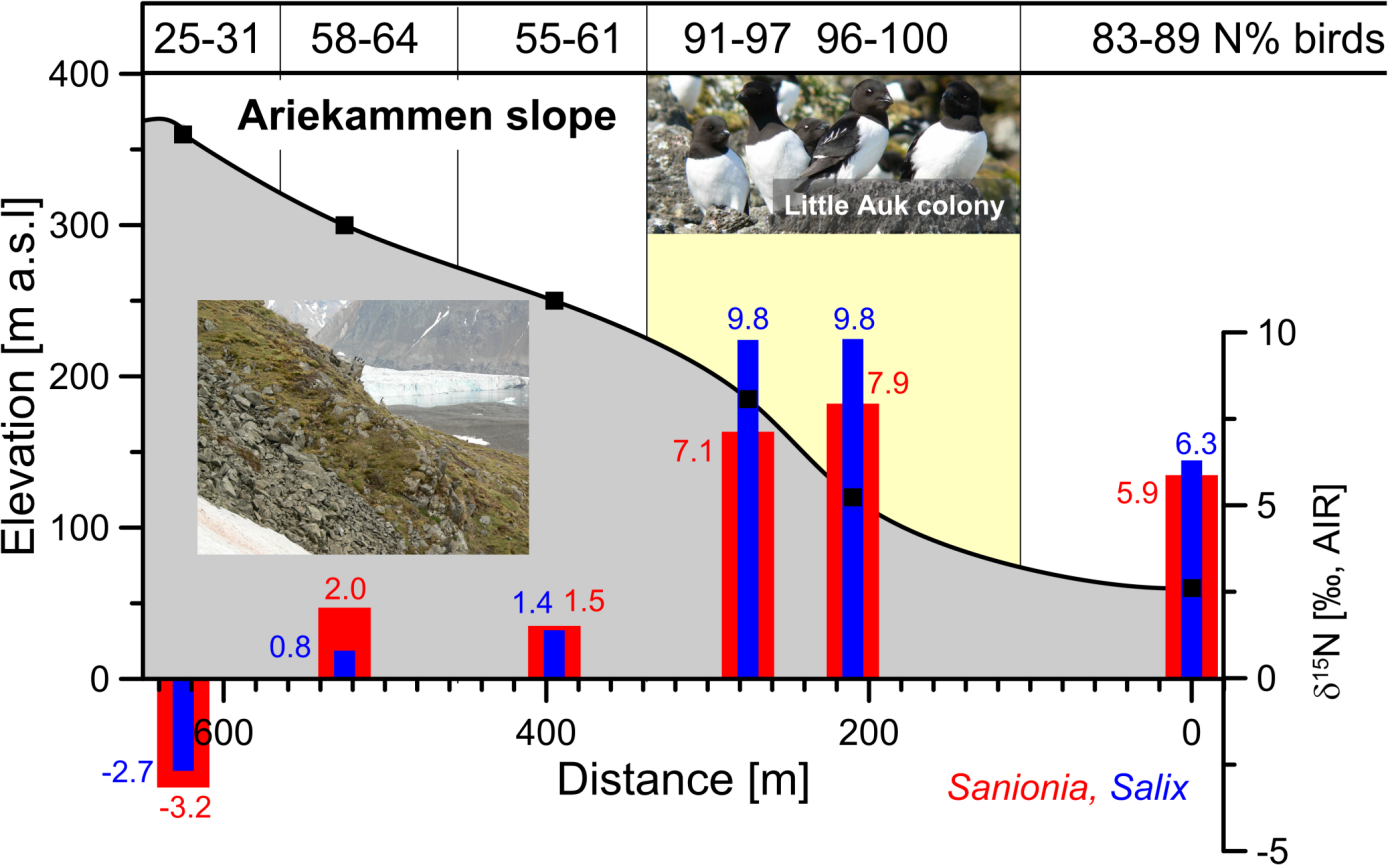
The stable nitrogen isotope composition of a moss (*Sanionia uncinata*—blue) and vascular plant dwarf willow (*Salix polaris*—red) collected on the Ariekammen slope at the study area Hornsund, Svalbard (sampling locations are marked with black squares on the slope grey slope cross-section). Contribution of Bird-originated N (N% in upper frame) is estimated based on the *δ* ^15^N signature of *Sanionia* and *δ* ^15^N of sources ±0.5‰. Photos by G. Skrzypek.

The ~625 m long altitudinal transect at Ariekammen slope (60–360 m a.s.l.) was used in this study to test the direct response of *δ* ^15^N of plants to different bird N-loads below and above the Little Auk colonies (Fig 3). Two plants, the moss *Sanionia uncinata* and *Salix polaris*, were collected from six locations at ~50–60 m altitudinal intervals. The transect started below the colony and extended through the colony area and the zone of circling flights of Little Auks (the area where birds fly in flocks around the colony, when frightened away by predators), and ended above the colony on the mountains ridge, where birds rarely flew.

### Stable isotope and chemical analyses

Samples of fresh plants (~5 g) were soaked in deionized water (DI), rinsed, and quickly dried at 50°C. Bird feces and soil samples were not treated but dried at 50°C. Snow samples containing *Chlamydomonas nivalis* were thawed, separated from dust, filtered and then 1 L of deionized water was passed through the quartz filter with samples to remove inorganic N. All samples were homogenized by grinding in a ball-mill to fine powders. Plant, soil, and feces samples were analyzed for *δ* ^15^N at the West Australian Biogeochemistry Centre, School of Plant Biology, The University of Western Australia, using a continuous flow analytical system consisting of an elemental analyzer Flush 1112 coupled with a Delta V Plus mass spectrometer via Conflo IV (Thermo-Finnigan, Bremen, Germany) [41]. Stable nitrogen isotope compositions were reported in the standard *δ-*notation following multi-point normalization using international reference materials provided by International Atomic Energy Agency (IAEA-N1, IAEA-N2, USGS32) and laboratory standards [42]. The uncertainty associated with stable isotope analyses for δ^15^N was not more than 0.10‰ (1 standard deviation). Samples for *δ* ^15^N and *δ* ^18^O in NO_3_ in water were analyzed as N_2_O on a GasBench II coupled with Delta V Plus (Thermo Scientific, Bremen, Germany) in the Stable Isotope Core Laboratory of Washington State University using the “denitrification method” [43, 44]. The results were normalized to VSMOW and AIR international scales using a multiple-point normalization method based on primary reference materials IAEA-N3, USGS32, and USGS34. The analytical combined uncertainty was <0.5‰ for *δ* ^15^N and *δ* ^18^O.

Water samples were filtered (0.45 μm) and analyzed for NH_4_^+^ and NO_3_^–^ by ion chromatography (Metrohm IC 761 Compact, Herisau, Switzerland). Soil samples, immediately upon collection, were sorted manually to remove living plants, and homogenized. Soil mineral N was extracted using 100 mL 1M KCl (for ammonium) and deionized water (for nitrates) [45]. The solution was filtered (0.45 μm) and analyzed for ammonium by the FIA method (FIA compact, MLE GmbH, Dresden, Germany) and nitrates as water samples.

### Isotope mixing model

The stable nitrogen isotope mass balance mixing model was used to estimate the contributions from the three major N-sources. The calculations followed the IsoSource algorithm designed by Phillips and Gregg [46] based on *δ* ^15^N of the mosses *Sanionia uncinata* and *Racomitrium lanuginosum*, which access the N deposited on their surfaces because of ectohydric nature of water and nutrient uptake [34]. The results were calculated as a mean percentage and a range fulfilling the mixing criteria for three N-sources and *δ* ^15^N. For tundra locations with only two confirmed N-sources, exact ratios based on mass balance model were calculated. The statistical significance of linear regressions models for the elevation transect was assessed based on the p-value for the F-test as a part of ANOVA analysis at the 95% confidence level. The unpaired t-Test has been used to test the significance of differences between means while comparing δ^15^N values for different tundra locations and N-pools.

## Results and Discussion

### *δ* ^15^N signature of three major N-sources for tundra vegetation

Different plants growing in the same location may access different N-pools, depending on their environmental adaptations. However, analyzing ectohydric mosses (SAN and RAC) reduces possible number of scenarios because these mosses mainly use N deposited on their surfaces. Bryophytes usually absorb NH_4_ more easily than they absorb NO_3_, but can also use organic forms of N (amino acids, especially glycine) if preferred forms are not available [47]. However, in N-limited environments, all available N is likely to be used.

#### *δ* ^15^N signature of bird-originated N

The first N-source *δ* ^15^N signature, initial bird-originated N, was determined directly from feces as 8.1±0.5‰ (Fig 2), what is within the range reported for the Little Auk’s preferred food, *Calanus glacialis*, in Kongsfjorden, NW Spitsbergen (7.0±0.9‰ [48]). Taking into account the 2–3‰ isotope fractionation due to trophic level, this value is also consistent with values obtained for samples of blood (11.1±0.3‰ [49]), muscle (11.2‰ [48]) and feathers (11.4±0.3‰ [50]) collected from Little Auks. However, the signature of bird feces is not necessarily a direct reflection of the *δ* ^15^N of N utilized from this source by mosses. Therefore, we also obtained indirect values that represent the N available for mosses from bird feces by determining the *δ* ^15^N of the cryophilic green alga *Chlamydomonas nivalis* growing on snow directly within the bird colony [51]. This value for *C*. *nivalis* (8.1±0.3‰ n = 6) was not significantly different (p = 0.85) from the *δ* ^15^N value in the bird feces (8.1±0.5‰). *C*. *nivalis* does not have the ability to fix atmospheric N_2_ and because as mosses prefers NH_4_ over NO_3_, its signature directly reflects the total *δ* ^15^N of atmospheric deposition proportional to NH_4_:NO_3_ ratio available for mosses [52]. In contrast, nitrates dissolved in the water of creeks below the colonies had more positive *δ* ^15^N(NO_3_) values (12.9±1.3‰; tundra T-WTM; see Table 1 and S3 Table). These values are unlikely to represent the original signature of the N-source delivered to tundra surface and used by mosses. Microbial activity in soil, even during the cold Arctic summer, is expected to be significant and may modify the initial signatures by enriching the remaining substrate in ^15^N, particularly during ammonia volatilization [53, 54].

In general, the extent of fractionation depends on the size of the N-pool. Hence, in N-limited systems, the fractionations are minimal [22]. For that reason, the possible ^15^N-enrichment is essentially restricted to bird colonies only. The *δ* ^18^O(NO_3_) in creek water (–5.7±0.7‰) also confirms that these *δ* ^15^N signatures were modified in soils but not in creeks, as *δ* ^18^O(NO_3_) was not statistically different (p>0.6) from the signature observed in a few low volume drizzles that occurred during the sampling period (~–6.00‰). It was also significantly different from atmospheric origin NO_3_ (~60‰, p<0.001), creek water (–10.1±1.2‰, p<0.001) and snow (–8.8 and –9.4‰, two snow pack samples next to the colony, p<0.01).

#### *δ* ^15^N signature of N originating from atmospheric deposition

The second N-source *δ* ^15^N signature, N originating from atmospheric deposition, is more difficult to average to a single variable for a mixing model, due to the significant episodic variability of N-deposition and generally very low concentrations [2]. The *δ* ^15^N values for Arctic NH_4_ and NO_3_ deposited with snow are low and variable [55]. The stable isotope composition of NO_3_ in snow from NW Spitsbergen (Ny-Ålesund, Kongsfjorden) was reported as –8.6±1.0‰ (*δ* ^15^N) and +70.8±3.0‰ (*δ* ^18^O) [53]; but no values have been reported for rainwater or NH_4_ because of extremely low concentrations. Rainwater samples collected during the present study also contained extremely low concentrations of NO_3_ and NH_4_ (<0.001 mg/L), which prevented precise analyses using the adopted precipitation method with preconcentration on resin [56]. Only one measurement using the “denitrification method” was successful. For that reason, the single obtained value of *δ* ^15^N –4.5‰ and *δ* ^18^O +60.5‰ cannot be considered as representative. Nevertheless, the *δ* ^18^O of this sample confirms the genuine atmospheric origin of this nitrate [22]. In the current study, the atmospheric N-source *δ* ^15^N signature was based on indirect measurements. The value of –6.2±1.1‰ obtained for *Polytrichum* and *Sanionia* mosses growing on mineral soils far from the bird colonies (the Tuva nunatak) was very likely to reflect an N-source that was derived exclusively from atmospheric deposition. These values also reflect the weighted mean of NO_3_ and NH_4_ signatures, reflecting the proportion between these ions in precipitation (Fig 2). Similar values were confirmed for *C*. *nivalis* growing on clean snow far from the bird colonies (–5.4±0.5‰). The possibility of additional contributions (e.g., from occasional bird visits or atmospheric N_2_-fixation) cannot be fully excluded in the sampling locations, so the lowest observed value of –7.5‰ as the N-deposition source was adopted as *δ* ^15^N of N atmospheric deposition in further models. Similar values were reported for rainfall in pristine areas worldwide (e.g., –7.5 to –5.5‰ [57, 58]).

#### *δ* ^15^N signature of atmospheric N_2_-fixation

In this study, *δ* ^15^N of the third N-source, atmospheric N_2_-fixation in the tundra environment, was determined by analyzing moss-cyanobacteria associations and *Nostoc* heterocysts. The obtained values ranged between –1.3 and 0.2‰, with a mean of 0.4‰ (n = 12, Fig 2). This mean value of 0.4‰ was used as a signature of the N originated from primary atmospheric N_2_-fixation. These values were in agreement with the general range reported earlier; the *δ* ^15^N of nitrogen originated from atmospheric N_2_-fixation varies according to Kendal [22] in a narrow range between –3 and 1‰, with an average fractionation around –0.52‰ for microorganisms in the soil [24], causing isotope fractionation around –0.25‰ only [59].

N_2_-fixation by cyanobacteria does not occur in all types of tundra and it should not be considered as an N-source everywhere. N_2_-fixation is an energetically expensive process and is very sensitive to inorganic N-inputs, so that when cyanobacterial N needs are satisfied by high NO_3_ or NH_4_ input, less N_2_ is fixed [60]. Therefore, N_2_-fixation does not contribute to an N-pool in an N-rich environment where it is inhibited by high N-concentration from bird feces, and so was not considered as an N-source at the T-OK and T-WTM locations. Soil moisture is one of the most important environmental factors controlling the N_2_-fixation activity of cyanobacteria-moss associations [61]. Therefore, this N-source is likely to have limited significance in dry T-MPN and T-KP tundra types, where *Nostoc* nodules or moss-cyanobacteria mats were not observed. However, both RAC and SAN commonly form associations at a more discrete cellular level [61, 62]. Heterocystous cyanobacteria associated with mosses [5] may contribute significantly to the formation of an N-pool through the process of N_2_ fixation [8, 63]. For this reason, all three N-sources (including atmospheric N_2_-fixation) were considered for these types of tundra.

The advantage of the direct analysis of *δ* ^15^N in N-sources is that the isotope signature of delivered N is precisely identified; however, it will not necessarily be fully reflected in plant tissue if isotope fractionation occurs. The use of algae or plants exposed to only one dominant N-source as a tracer reflects directly assimilated N regardless of its form. However, in natural field conditions, exclusion of other N-sources that can potentially contribute to the pool accessible to a plant is not always possible. Given all these precautions, the combined approach used here—that compared the *δ* ^15^N of N delivered to the tundra surface with the *δ* ^15^N of plants—allowed a more accurate determination of isotope signatures for the main N-sources for tundra.

### Spatial distribution–altitudinal transect across a bird colony

The *δ* ^15^N along the elevation transect at Ariekammen slope (60–360 m a.s.l.) varied from 9.8‰ (*Salix*) and 7.9‰ (*Sanionia*) in the middle of Little Auk colony to –2.7‰ (*Salix*) and –3.2‰ (*Sanionia*) on the top of the mountain ridge (Fig 3). This distribution of *δ* ^15^N values was consistent with the expected feces deposition, given the colony distribution and the flying patterns of the birds (Fig 3). However, *δ* ^15^N values were consistently 20% higher for *Salix* than for *Sanionia*. Nevertheless, the *δ* ^15^N values for both plants were strongly correlated (r^2^ = 0.95 p<0.001, Figure B in S2 File), suggesting that the consistent pattern does reflect the loads from bird feces deposition. The observed differences between the two plants can be explained by accessibility to different N-pools, with constant differences in magnitude across the gradient.

Most vascular plants derive N mainly from the soil. In contrast to *Salix* (dwarf willow), *Sanionia* moss lacks roots, which limits its access to the soil N-pool. Hence, *Sanionia* acquires most of its nutrients by direct adsorption through the leaf surfaces. In contrast, dwarf willow, as a vascular rooted plant, has access to deeper soil N. Soil tends to have higher *δ* ^15^N values, due to fractionation during denitrification and ammonia volatilization. Therefore, rooted dwarf willow had a higher *δ* ^15^N value compared to *Sanionia*. This pattern suggests that mosses reflect more directly the current deposition pattern while vascular plants reflect the soil N-pool, which can be modified by post-depositional processes. Therefore, the values obtained from *Sanionia* and *Racomitrum* (but not vascular plants) were used to estimate the direct input from different N-sources in various types of tundra.

The Ariekammen colony is inhabited by >22,000 breeding pairs of Little Auks for three summer months between the beginning of May to mid-August [60]. Production of feces by the Little Auk colony during one breeding season is estimated at 59.6 g/m^2^ directly on the colony site, 25.1 g/m^2^ in the circular flight zone around the colony, and 0.60 g/m^2^ in the tundra between the colony and the sea [9]. In general, these estimates are consistent with rough estimates of up to 1.2 g/m^2^/day of dry feces mass deposited directly at the colony site and up to 0.5 g/m^2^/day in a 200 m stretch between the colony and the sea [15]. These estimates, which were based on 20–36 h exposures of plastic sheets and calculating deposition based on feces coverage using digital photography, reflect the total deposition; they do not necessarily translate directly to the N that was bioavailable and assimilated by the tundra plants. Feces deposition is subject to quick washout due to precipitation, snow melt, and the highly porous Arctic soils, which drain easily on steep hillsides. The advantage of a stable isotope mixing model is that it allows calculation of the direct input from different N-sources to the plants. Hence, not surprisingly, the contribution of the bird-N to moss growth was 91–100% at the colony site, 83–89% below the colony, and 25–61% between the colony and the mountain ridge, assuming a two-end members N-isotope mixing model (*δ* ^15^N of atmospheric N-source = –7.5±0.5‰ and bird N-source = +9.1±0.5‰).

### N-sources in various types of tundra

The stable nitrogen isotope composition of tundra plants in the Fuglebekken catchment showed a wide variability with *δ* ^15^N values ranging from –5.45‰ (lichen *Cladonia rangiferina*, (L.) Weber ex F.H. Wigg. T-MPN) to 14.24‰ (*Tetraplodon mnioides*, (Hedw.) Bruch & Schimp., T-WTM), which reflected different contributions from the three major N-sources and different N-pools available to different groups of plants (Figure B in S2 File, Tables A and B in S3 File, S1 Table). The mosses (*Sanionia uncinata* and *Racomitrium lanuginosum*) mainly reflected the signatures of deposition on the tundra surface and therefore the main N-sources only (rather than the soil, as reflected by vascular plants), and varied in a *δ* ^15^N range from –4.82‰ (T-PG) to 13.47‰ (T-WTM).

**Fig 4:**
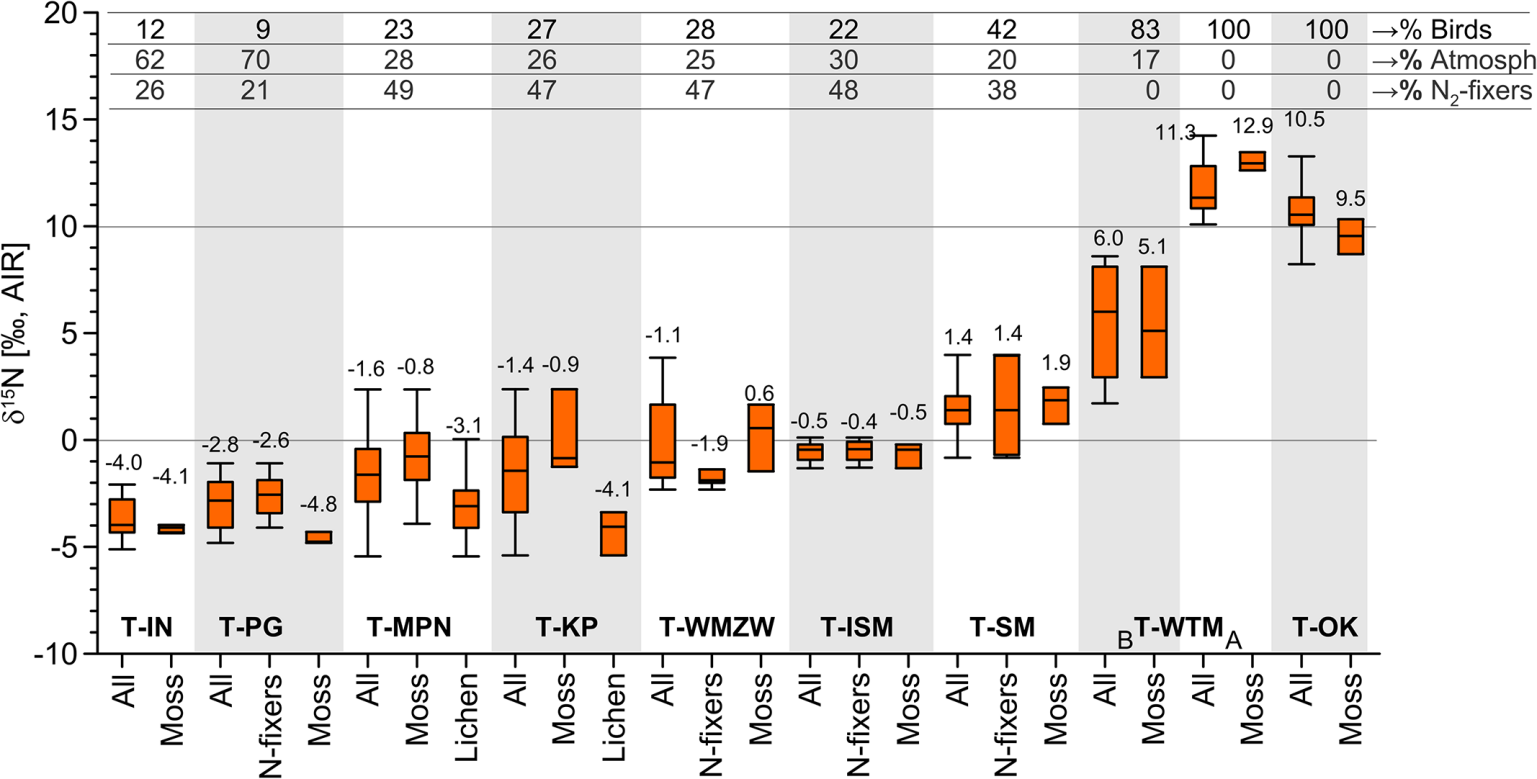
The stable nitrogen isotope composition of dominant species in various/particular types of tundra in the studied area from the lowest to the highest *δ* ^15^N mean values (refer to Fig 2 and Table 1). The Box-Whisker plot shows minimum and maximum (caps at the end of each box), the lower and upper quartiles (orange box), and the median (line inside the box). The numbers in the top table reflect the estimated relative mean contribution from the three major N-sources to total N-pool, separately for each type of tundra. The estimate of N-contributions based on *δ* ^15^N, botanical, and algological studies, and is given as an N-percent incorporated by plants from each N-source. “All”—refers to mean for all sampled plants from each tundra location as in S1 Table. Tundra type symbols/ abbreviations–see Table 1.

The N-source in Geophytic initial (T-IN) and Patterned-ground (T-PG) tundra was not significantly different (p = 0.16) and was dominated by atmospheric deposition, at 42–79% (T-IN) and 53–84% (T-PG). The *δ* ^15^N values of mosses were close to those of other coexisting plants, suggesting a unified N-pool for all plants (Fig 4). The bird contribution cannot be truly excluded and likely ranged from 0–26% (T-IN) and 0–21% (T-PG). However, in a three-source mass balance model, this contribution can be compensated/masked by primary N_2_-fixation, which possibly varies from 0 to 58% (T-IN) and from 0 to 47% (T-PG). The higher *δ* ^15^N of bulk soils, at 2.42‰ (T-IN) and 3.22‰ (T-PG), was not reflected in mosses, indicating that, as expected, soil N is not available for the moss species examined here (S1 Table). Both the T-IN and T-PG types of tundra vegetation show that N may be the main limiting factor for net primary productivity at the initial stage of primary succession [64, 65]. The estimated N_2_-fixation rate in T-IN and T-PG was similar to general estimates for cyanobacteria-moss associations (2–58% of N) reported from Arctic ecosystems elsewhere [7, 8].

Two types of dry tundra, Lichen-prostrate (T-KP) and Epilithic moss-lichen (T-MPN) tundra were not significantly different from each other (p = 0.76) and show that the potential contribution of primary N_2_-fixers can be relatively high, at 47% (0–100%) in T-KP and 49% (0–100%) in T-MPN. Lichens are unlikely to contribute to the bioavailable pool as the dominant photobionts in the observed lichens (*Parmeliaceae*, *Cladoniaceae* and *Lecanorales*) are green algae (*Chlorophyta*) that lack the ability to fix atmospheric N. Therefore, the contribution can instead be linked with epiphytic associations with cyanobacteria. The load of N is received from bird feces and atmospheric deposition, varying in the landscape at 0–51% (T-KP) and 0–48% (T-MPN) (Fig 4, Table 1). However, a large range of spatial variation was observed, which likely reflects random accidental distributions of bird feces. The locations were frequently elevated above groundwater level, so the relatively high biomass production can be attributed to the relatively high N-loads delivered directly by birds on the tundra surface and by associated cyanobacteria. The soil N pool, similar to that recorded for T-IN and T-PG, was not accessible to the mosses and shows a much higher *δ* ^15^N signature than was seen for vascular plants, at 2.79‰ (T-KP, p<0.001) and 3.06‰ (T-MNP, p<0.001).

The *δ* ^15^N in plants from Flow water wet moss tundra (T-WMZW) showed a similar range of variation to that seen in T-KP (p = 0.92) and T-MPN (p = 0.68), and it also reflected the location rather than the general environmental conditions, with *δ* ^15^N ranging from –2.32 to 3.85‰. In T-WMZW, the presence of primary N_2_-fixing cyanoprokaryota was confirmed by microscopy and the *δ* ^15^N for the cyanobacteria-moss association varied at around –1.88‰ and for the algal thallus at around –1.85‰. N_2_-fixation likely dominated the available N-pool (mean 47%, 0–98%). This N-contribution from primary atmospheric N_2_-fixation is in good agreement with previous studies identifying that N_2_-fixation accounts for up to 80% of total landscape annual N inputs in wet type of tundra [4]. In contrast, contribution of the bird-originated N was low, at ~28% (2–52%), and similar to atmospheric deposition, at ~25% (0–53%).

A high input from N_2_ primary fixation, similar to that seen for T-WMZW, was characteristic of two other types of wet tundra: Initial cyanobacteria-moss (T-ISM: ~48%, 0–100%) and Snowbed cyanobacteria-moss (T-SM: ~38%, 0–81%). However, because of possible variable contribution from all three N-sources, a bird contribution of ~22% (0–46%, T-ISM) and ~42% (19–62%, T-SM) cannot be excluded. Similarly, as seen in the other types of tundra described above, the soil N-pool had much more positive values than those in plants, at 3.51‰ (T-ISM, p<0.001) and 3.08‰ (T-SM, p<0.001) (S2 Table).

The highest bird N-loads were confirmed for Wet moss tundra (T-WTM) and Ornithocoprophilous tundra (T-OK), where contribution of the bird originated N accounted for up to 100% of total N used by plants. This very high bird N-load means that the contribution from atmospheric deposition is negligible. The T-OK situation is relatively simple, as *Sanionia uncinata* had a value of 9.50‰, which was close to the upper limit of *δ* ^15^N observed in bird excrement (7.15–9.04‰). The assemblage of dominant species in the Wet moss tundra (T-WTM) slightly varied depending on the distance from the colony and mean *δ* ^15^N were significantly different (p = 0.007); therefore, it was divided into two subgroups (A and B on Fig 4). The moss *δ* ^15^N in T-WTM-B varied between 2.94‰ and 8.11‰, reflecting an average of ~83% for the bird-N contribution (67–100%) and 17% for atmospheric N deposition (0–33%). In contrast, an exceptionally high *δ* ^15^N was observed for T-WTM-A, where *Sanionia uncinata* moss (12.62–13.47‰) as well as all other plants had *δ* ^15^N values ranging 10.08–14.24‰ (Fig 4). These high *δ* ^15^N values for mosses (above that observed for fresh bird feces) might suggest that N from highly decomposed soils is exceptionally available to mosses at this particular location. T-WTM-A is a wet moss tundra located directly below the bird colonies and it is frequently waterlogged with water that flows through the soils and regolith heavily impacted by feces deposition in the colonies.

The T-WTM-B is supplied by water that drains directly from the Little Auk colonies; it was characterized by a very high water level and was frequently waterlogged during growing season. The *δ* ^15^N of bulk soil N in this location varies in the range of value reported for plants, i.e. 12.02–13.94‰. The bird feces deposition on T-WTM-A is similar to that seen for T-WTM-B. However, T-WTM-A is located further from the colonies and because of topography, it is not supplied directly by water discharged from the bird colonies. In general, high rates of ammonia volatilization caused by biological decomposition of bird droppings, which constitute a large N-pool in these types of tundra, may cause fractionation leading to ^15^N-enrichment of the remaining N in the ecosystem [22, 66]. Extremely high N-loads that significantly exceed demand may also result in some fractionation during N-metabolism due to efflux [20]. For these reasons, the *δ* ^15^N of mosses in T-OK and T-WTM exceeds the values of the dominant N-source, the bird droppings.

In addition to Little Auks, other vertebrates also deposit excrement on the tundra surface. Jakubas et al. [67] estimated that herbivores, such as geese (*Anser brachyrhynchus* and *Branta leucopsis*) and reindeer (*Rangifer tarandus*), deposit ~0.2 g/day/m^2^ and ~0.02 g/day/m^2^ of excrement during the growing season, respectively. However, although this N-load is not negligible, it actually cannot be viewed as an external input to the tundra. Rather, it represents a recycling of N from the tundra, because these birds and reindeer graze on the tundra and return what was consumed. The extent to which the tundra grazers may influence the stable nitrogen mass balance and increase uncertainty in the N-budget is unknown. However, taking to account the relatively small mass of N retained in animal bodies compared to the total pool of N and that both urine and feces were deposited back to tundra, this effect is likely to be limited.

Laboratory studies have reported isotope fractionation between the stable nitrogen isotope composition of plant tissues and solution; however, the consensus is that isotope fractionation in most cases occurs only when plant N-demand is much lower than supply [68–70]. Fractionation may occur if excessive leakage of inorganic N occurs after uptake from roots, which is likely to happen in N-rich environments only [69] such as the Ornithocoprophilous tundra (T-OK). Nevertheless, in most cases, differences in access to different N-pools, and not isotope fractionation itself, are the main reasons for the observed *δ* ^15^N differences in plants that coexist in each location [70]. However, differences in nitrogen assimilation and transfer processes cannot be excluded and add extra uncertainty to the overall estimations presented here [18, 71, 72].

## Conclusions—Nitrogen Budget in the Catchment

The contributions of the three major N-sources (birds, atmospheric deposition, and N_2_-primary fixation) vary in wide range across the studied catchment. The total N-budget in the catchment depends on N-loads received from birds in particular types of tundra and on the size of the areas covered by these types of tundra. On average, the relative contribution from the three N-sources in the Fuglebekken catchment is as follows: 36% from birds, 38% from atmospheric deposition, and 26% from atmospheric N_2_ fixation.

Seabirds, like Little Auks, provide large amounts of N but this supply is highly localized within the breeding colonies. Therefore, some tundra types are N-saturated (e.g., T-OK, T-WTM-B), while other areas (e.g., T-PG, T-IN, T-ISM) receive negligible amounts of the bird-originated N. However, even those types of tundra (e.g., T-MPN and T-KP) that are not directly affected by runoff of water from colonies have a substantial contribution from the bird N-load due to the considerable amount of feces in the flyover zone. The tundra directly supplied with the bird-N (T-OK and T-WTM-B) constitutes only 21% of the total tundra area in the studied catchment, and yet, on average, 21% to 51% of N used in the whole catchment is of bird origin (mean 36%). Therefore, bird feces serve as an essential N-source in the tundra and contribute significantly to biomass production and therefore carbon sequestration.

Climate change, both currently observed and that predicted for the future in polar regions, [73] may have serious consequences for the structure and functioning of the terrestrial part of the High Arctic ecosystems. Large colonies of planktivorous Little Auks are located inland, on mild mountain slopes and talus, usually a few kilometers from the shore [74]. They strongly influence large adjacent land areas by enriching the tundra with great amounts of feces. In contrast, the colonies of the piscivorous seabirds, like guillemots (*Uria* sp.), situated on rock cliffs close to the shore, have a much more limited range of impacts on the tundra N-pool because of the short distance to the sea and the rapid washing-out of the biogenic nutrients (feces) back to the sea [75]. Climate change is predicted to result in worsening feeding conditions for planktivorous seabirds and reductions in their populations, while favouring piscivorous species [11, 14].

This type of scenario predicts that large areas of tundra currently supplied with nutrients of marine origin from plankton-eating seabird colonies may disappear. In contrast, smaller patches of Ornithocoprophilous tundra that exist under the cliffs inhabited by piscivorous seabirds, with low total production, will predominate but will not extend into areas previously inhabited by planktivorous species. Consequently, these smaller areas of highly productive tundra will produce less plant biomass and will limit soil development and decomposition processes, which may also have feedback effects on soil formation processes and consequently on plant succession. These smaller patches of tundra also may be unable to support the current populations of large herbivores, such as geese and reindeers [11, 14, 15, 67, 76].

Atmospheric N-deposition is more or less the same over the whole catchment area. However, the retention time of this N depends on the retention of precipitation and runoff, which in turn is associated with tundra type and microtopography, as concluded recently by Stewart et al. [4]. The use of this source of N is likely to be more efficient in areas where water is retained longer (e.g., T-IM, T-ISM). The dryer areas that are elevated above the surface water table (e.g., T-KP), instead experience a quick flush-through, and whatever N which is not used immediately is flushed down with the runoff. Atmospheric deposition constitutes 20–56% of the N (mean = 38%) used by plants in the whole catchment, mainly because of the large areas covered by T-IN tundra, where atmospheric deposition is the dominant N-source.

Primary N_2_-fixation depends on microhabitat type and the presence of cyanobacteria, which are capable of atmospheric N_2_-fixation but only actually acquire atmospheric N_2_ if other N-sources are unavailable. The presence of N_2_-fixing cyanobacteria was confirmed by microscopy in T-ISM, T-PG, T-SM, T-IN and T-WMZW. Cyanobacteria can also be present in T-KP and T-MPN; however, their contribution to the N-pool available for plants is highly variable. On average, approximately 26% of the N used by plants in the whole catchment originates from primary N_2_-fixation (0–56%). The relative contributions from different N-sources reflect N-loads and determine tundra vegetation types.

The stable nitrogen isotope composition of mosses was successfully used to estimate the contribution of different N-sources in tundra, even in locations where more than two sources contributed to total N. The δ^15^N values indicate the ratio between the amounts of N acquired by plants from different N-sources, and not necessarily the actual deposition on the tundra surface, which can be higher and partially removed by runoff. The N-pool available for different plants is not necessarily the same because of differences in physiology, N-acquiring strategies, and preferences. Additional uncertainty can arise from possible fractionation due to ammonia volatilization, nitrification and denitrification and different δ^15^N signatures of different N-bearing chemical compounds. Despite these constraints, δ^15^N gives a good estimate of N-source contributions that would otherwise not be possible using conventional techniques, thereby allowing quantitative determination of relative N-mass balances in tundra.

This study has been conducted on the scale of a single catchment, which does not necessarily represent all tundra habitats. However, the methodological approach used here can be easily applied to investigate contributions from different N-sources to various types of tundra elsewhere. The estimated ratios between different N-source contributions to various types of tundra can be also useful for upscaling to larger areas if types of tundra are to be mapped.

## Supporting Information

S1 File Figure A and dataset—temperature and precipitation in Hornsund in sampling season 2011 in relation to the 30-year mean (1979–2010) based on GLACIO-TOPOCLIM Database. (XLS)

S2 File Figure B and dataset—relationship between δ^15^N of a moss (*Sanionia uncinata*) and a vascular dwarf plant (*Salix polaris*) from the same locations experiencing different loads of N-deposition from bird feces but similar atmospheric N-deposition. (XLS)

S3 File Tables A and B—the stable nitrogen isotope composition of N-sources. (XLS)

S1 Table The stable nitrogen isotope composition of plants in ten tundra types. (XLS)

S2 Table The stable nitrogen isotope composition of soil in ten tundra types. (XLS)

S3 Table The stable nitrogen and oxygen isotope composition in water. (XLS)
